# Advancements and trends in agricultural veterinary education research: a text mining and network analysis approach

**DOI:** 10.3389/fvets.2025.1709176

**Published:** 2026-01-09

**Authors:** Liang Huang, Hairong Wu, Juhua Peng, Jinsi Liu

**Affiliations:** 1 School of Political Science and Public Administration, Wuhan University, Wuhan, China; 2 School of Marxism, Central China Normal University, Wuhan, China

**Keywords:** agricultural veterinary education, complex networks, knowledge graph, text mining, visualization

## Abstract

**Introduction:**

With the deepening integration of agriculture and veterinary medicine, improving the professional competence of grassroots veterinarians and livestock farmers has become particularly important. Agricultural veterinary education plays a crucial role in promoting sustainable agricultural development and cultivating a skilled veterinary workforce. However, current research on agricultural veterinary education is relatively scarce. Therefore, this paper explores the current state of research in this field and identifies its future trends, aiming to provide suggestions and references for research in agricultural veterinary education.

**Methods:**

This study systematically reviewed the literature in this research field using text mining, knowledge graphs, and social network analysis, with a focus on the research trends and challenges in agricultural veterinary education. Through comparison of literature from different countries and regions, the differences and characteristics of agricultural veterinary education research in various countries were revealed. Particular attention was paid to educational content, research priorities, and international cooperation and knowledge exchange.

**Results:**

Research on agricultural veterinary education generally focuses on teaching methods, development strategies, and vocational training in agriculture. In China, the emphasis tends to be on aligning national policies with the broader agricultural development goals. On the other hand, other countries place more importance on practical educational approaches, particularly focusing on improving the skills of veterinarians and livestock farmers at the grassroots level. However, research on agricultural veterinary education at the secondary school level is still limited. Additionally, there is a growing need for more interdisciplinary research, especially in areas such as agricultural environmental veterinary education, which requires a deeper exploration.

**Discussion:**

Future research should strengthen interdisciplinary collaboration to promote the comprehensive development of agricultural veterinary education. In particular, attention needs to be paid to education programs for grassroots veterinarians, and the curriculum should be improved by incorporating modern agrarian concepts such as environmental protection and circular agriculture. Furthermore, increasing practical components in agricultural veterinary education and enhancing teacher quality and overall educational standards will contribute to the advancement of agricultural veterinary education globally.

## 1 Introduction

As an interdisciplinary field, agricultural veterinary medicine is based on biology and encompasses a multi-level education system, ranging from secondary vocational schools to junior colleges and universities. It also provides training for livestock farmers and grassroots veterinarians, integrating agriculture and veterinary medicine to enhance their skills and expertise. While agricultural veterinary education shares a standard core structure based on biology, significant differences exist between veterinary education systems and degrees across countries. In some countries, veterinary training may place a greater emphasis on academic research, focusing on theory and scientific investigation, with students often earning master’s or doctoral degrees. In other regions, veterinary training is more practice-oriented, with students typically earning associate’s or bachelor’s degrees in veterinary science. Their primary responsibilities focus on animal care, disease prevention, and supporting agricultural production in rural areas. These differences in education systems impact the scope and depth of veterinary services, as well as the degree of integration between agriculture and veterinary medicine (1). The term “grassroots veterinarians” refers to veterinary professionals who are typically not trained at the graduate level and whose education primarily focuses on practical, hands-on experience rather than extensive academic research. Grassroots veterinarians usually hold associate’s or bachelor’s degrees in veterinary medicine and play a key role in livestock health and welfare at the local level. Their work focuses on animal care, disease prevention, and agricultural support in rural areas, where practical expertise is essential to improving productivity. Key areas of focus include animal disease prevention and control, optimization of livestock production, prevention and control of zoonotic diseases, enhancement of the agricultural production environment, and mitigation of public health risks related to agriculture. In the context of global agricultural sustainable development, the significance of agricultural veterinary education is becoming increasingly important. Agriculture is the fundamental industry of the national economy and the material foundation for ensuring people’s livelihoods. Only by ensuring the stability and efficiency of agricultural production can the challenges of food security and sustainability be effectively addressed (2). Additionally, developing the agricultural industry requires effective animal disease prevention and control, with zoonotic disease control being a crucial component. Thus, agricultural veterinary education has become a key tool for supporting agricultural growth, promoting livestock development, and controlling zoonotic diseases. With the acceleration of agricultural modernization, agriculture provides raw materials for various industries and a substantial workforce, contributing significantly to economic and social development. However, agricultural veterinary issues are becoming increasingly prominent. Farmers and grassroots veterinarians urgently need professional agricultural veterinary education and training to improve their response capabilities (3). One of the core objectives of agricultural veterinary education is to cultivate professional talents with a strong foundation in veterinary knowledge. This will enable farmers and grassroots veterinarians to effectively prevent and control animal diseases, optimize breeding models, and promote the sustainable development of animal husbandry, as well as the prevention and control of zoonotic diseases. Throughout agricultural development, agriculture has evolved from primitive to modern, increasing production efficiency and making the agricultural production environment more complex (4). In this process, agricultural veterinary education emerged and gradually became a crucial component in ensuring the sustainable development of agriculture (5).

China is the world’s third-largest country by land area, but it has the largest population. Compared to the United States, China’s population exceeds 1 billion. Land resources are scarce in some developing countries, making the importance of agricultural veterinary education even more significant. Developing modern agricultural veterinary education can effectively promote the healthy development of animal husbandry. This strengthens the ability to prevent and control zoonotic diseases, which is of great significance for ensuring a stable agricultural supply and maintaining public health security (6). As early as 1983, China’s State Council issued the “Notice of the State Council on the Report of the Ministry of Agriculture, Animal Husbandry, and Fisheries on the Conference on Agricultural Work in the Northern Arid Areas.” The document emphasized the need to pay close attention to agricultural veterinary education. In 1993, China promulgated the “Agricultural Law of the People’s Republic of China.” This law focuses on agricultural science, technology, and agricultural veterinary education, requiring governments at all levels to gradually increase funding for agricultural veterinary education. Although the text of the law has been revised and improved at different stages, agricultural veterinary education remains crucial to its content. In 2015, the General Office of the State Council of China issued the “Guiding Opinions of the General Office of the State Council on Promoting the Integrated Development of the Primary, Secondary, and Tertiary Industries in Rural Areas.”^1^ The document explicitly calls for developing a multi-type rural industry integration model, especially the coordinated use of existing resources to build agricultural veterinary education and social practice bases (7). Globally, agricultural veterinary education is being implemented in various countries. The United Kingdom once established many agricultural veterinary schools in Malaysia, aiming to spread advanced science and technology to multiple regions through agricultural veterinary education (8). Agricultural veterinary education in India during the 19th century played a crucial role in promoting scientific agricultural education, laying the groundwork for the institutionalization of agricultural veterinary education (9).

During the 1920s to the 1950s, the two world wars drew attention to the contributions of agricultural veterinary education to rural modernization. Professional farmers ensure that rural areas maintain economic vitality (10). The primary objective of agricultural veterinary education is to cultivate a cohort of agricultural veterinary professionals to meet the growing demand for agricultural veterinary talent in the current market (11). However, from the perspective of the entire agricultural veterinary talent market, students’ agricultural veterinary literacy needs improvement (12), highlighting the shortcomings of agricultural veterinary education. Agricultural environmental veterinary education provides a feasible solution. In addition to focusing on agricultural veterinary theoretical knowledge, incorporating more practical agricultural veterinary activities is even more critical. Practical teaching can compensate for certain shortcomings, stimulate students’ interest, and enhance agricultural veterinary practical skills (13). In promoting agricultural veterinary education, the professional literacy of instructors is also key to success. For this reason, it is necessary to fully integrate agricultural veterinary technology into classroom teaching (14). Moreover, agriculture faces significant global challenges, and sustainable agricultural development has become a widely accepted goal. Agricultural veterinary education is critical in cultivating professionals for a sustainable agricultural transition (3). By 2050, global food demand is expected to rise, increasing the likelihood of food crises. To address these future challenges, agricultural veterinary education has become a crucial means to expand the dissemination of agricultural veterinary technology (2).

Therefore, understanding the future trends of agricultural veterinary education is crucial. However, maintaining consistency in vocational education development is challenging due to significant disparities in economic development across countries and regions. In the face of population growth and climate change, the transformation and upgrading of agricultural veterinary education is inevitable. For most developing countries, agricultural veterinary education remains a weak link. Therefore, understanding the future trends of agricultural veterinary education plays a vital role in regional economic and social development. With the continuous development of precision agriculture, smart agriculture, and sustainable agriculture, veterinary education in agriculture must also undergo a corresponding transformation and upgrading. Agricultural veterinary education needs to adapt to these new technologies and concepts, which include not only skills enhancement but also an understanding of new agricultural ecosystems and corresponding strategies. In short, reform of modern agricultural veterinary education is imperative. As a precursor to agricultural veterinary education practice, academic research must address current shortcomings in agricultural veterinary education and provide practical and feasible solutions. However, current research on agricultural veterinary education is fragmented and lacks a clear understanding of its importance. This lack of knowledge about the current state of agricultural veterinary education development hinders the provision of practical guidance for governments and organizations promoting agricultural veterinary education.

## 2 Data sources and research methodology

### 2.1 Data sources

Agriculture is a fundamental source of livelihood for human society, and the promotion of agricultural veterinary education is undoubtedly crucial for its development. However, the advancement of agricultural veterinary education worldwide faces challenges and difficulties, prompting increasing interest in this field. This article systematically reviews global research on agricultural veterinary education. This paper utilizes data from two major databases: China National Knowledge Infrastructure (CNKI) and Web of Science (WoS). The reason for this approach is that this paper is based on research conducted in China while also expanding upon a global research perspective. Among Chinese scientific literature databases, CNKI is the most comprehensive and representative. Globally, WoS is the most authoritative and comprehensive. Therefore, choosing these two databases provides broad coverage of agricultural education literature in both Chinese and English.

**Figure 1:**
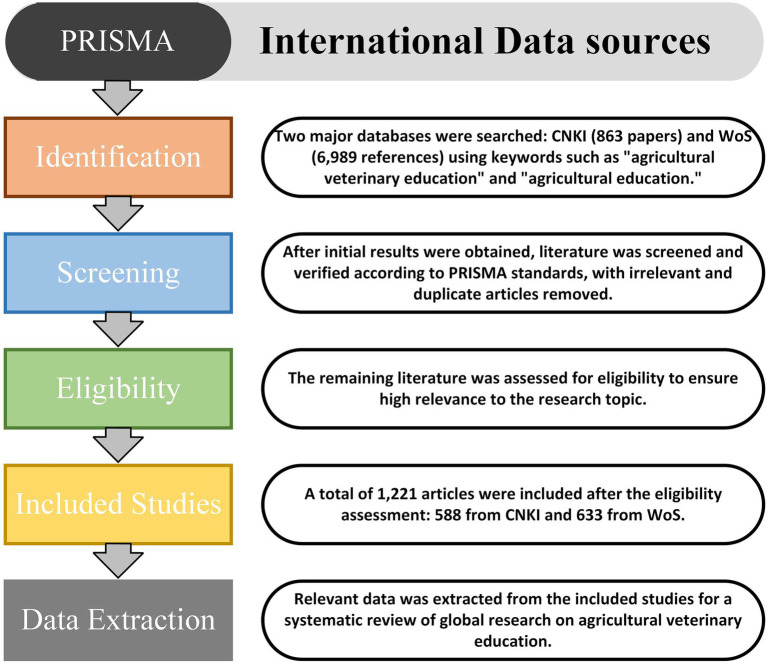
Literature screening process based on PRISMA standards.

Entering “Advanced Search” on the CNKI official website and searching for “agricultural veterinary education” and “agricultural education” yielded 863 relevant papers. Then, based on the PRISMA standard (Figure 1), after checking and verifying all the literature, irrelevant and duplicate literature were deleted, leaving 588 highly relevant articles. On the WoS platform, selecting Core Collection and ESCI-compliant sources, and entering “Agricultural Veterinary Education” and “Agricultural Education” as search terms, yielded 6,989 references. Next, referring to the PRISMA standard, after checking and verifying all the literature, irrelevant and duplicate literature were deleted, leaving 633 articles with high relevance.

**Figure 2:**
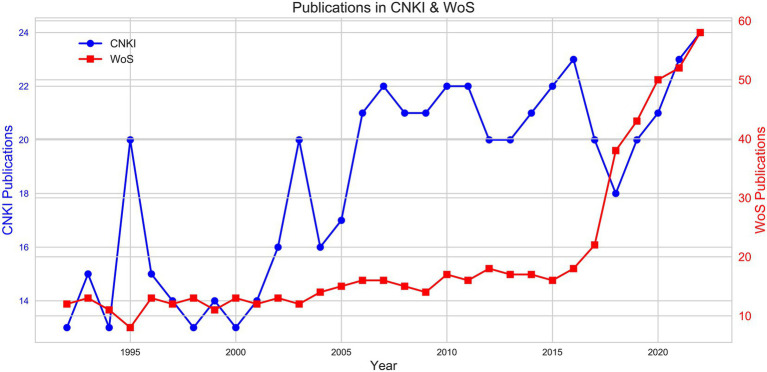
Statistical analysis of agricultural veterinary education publications.

After obtaining the final number of documents from the CNKI and WoS databases, the publication year of these documents is then tallied. This enables us to retrieve the number of publications on this topic for each year from both databases. Next, a dual-axis line graph is plotted using Python (Figure 2). Both CNKI and WoS showed a steady increase in the number of publications from 1992 to 2022. This indicates a gradual rise in global attention and research output on this research topic. The WoS saw a particularly significant increase between 2018 and 2022, demonstrating a rapid rise in international academic exchange and the growing international influence of this topic.

### 2.2 Research methodology

This paper employs text mining, knowledge graphs, and social network analysis to develop a quantitative framework for a literature review in agricultural veterinary education. By combining these methods, the literature on agricultural veterinary education can be analyzed more in-depth and systematically, revealing the internal laws and relationships within the field. Text mining is extracting potential knowledge and patterns from massive unstructured text data through technical means such as information retrieval, text analysis, and information extraction (15). Specifically, text mining involves the automated analysis of keywords, themes, and patterns in documentary data to reveal its underlying information structure. It not only helps identify common themes in a text but also reveals deeper meanings behind it through natural language processing techniques, such as word frequency analysis and cluster analysis. Systematic literature reviews can be effectively generated using text mining methods (16), especially by analyzing text corpora to construct topic models and trend graphs (17). In recent years, text mining methods have been widely employed in educational research, particularly in processing and analyzing large volumes of literature data, highlighting their unique advantages (18). Text mining is a powerful tool that can reveal the internal structure and associations within literature (19). However, in agricultural veterinary education, few scholars have applied it to the writing of literature reviews, which is also a significant innovation of this study. Based on text mining methods, knowledge graphs have been widely used in academic research as a powerful supplementary tool (20). Knowledge graphs can extract important information from structured or semi-structured data (74) and visually present graphically the relationships and knowledge networks between various data types (22). Specifically, it is a technology that uses nodes and edges to visualize knowledge, concepts, entities, and their interrelationships.

In addition, this paper innovatively introduces social network analysis methods (21), which mainly focus on the interactions and connections between different objects. Social network analysis is a technique used to study social structures and the relationships within them. It uses graph theory models to analyze the connections and influences between different individuals or organizations. By combining multiple technical means such as text mining, knowledge graphs, and social network analysis, this paper can conduct a more in-depth quantitative analysis of the literature in agricultural veterinary education, revealing research trends, hot topics, and potential research directions in this field. The principle is to treat a pair of words appearing in a text as a co-occurrence pair and count the number of co-occurrences of each word. The steps are as follows: First, the text data of Chinese and international literature on agricultural veterinary education is preprocessed to remove stop words and obtain a list of valid words. Next, a word co-occurrence network is constructed based on the order in which the words appear in the text. If two words appear at both the *i-*th and *j-*th positions, there will be an edge in the network from the *i-*th position to the *j-*th position, with the weight of the edge equal to the number of times the two words appear together. Finally, the in-degree and out-degree of each word are calculated: the in-degree is the number of edges connected to the word, and the out-degree is the number of edges originating from the word. To assess the role of different keywords in the entire network, this article uses betweenness centrality. If a node is on multiple shortest paths from various nodes, it indicates that it has a high betweenness centrality. In betweenness centrality, the absolute betweenness centrality is first calculated (Formula 2), from which the relative betweenness centrality can be further calculated (Formula 3). Here, it is assumed that there are $g_{\mathrm{jk}}$ paths between nodes *j* and *k*, and the number of paths between nodes *j* and *k* that pass through point *i* is represented by $g_{\mathrm{jk}}(i)$ . The ability of point *i* to control the interaction between the two points is defined by $b_{\mathrm{jk}}(i)$ (Equation 1).


(1) $$b_{\mathrm{jk}}(i)=g_{\mathrm{jk}}(i)/g_{\mathrm{jk}}$$

(2) $$C_{\mathrm{ABi}}=\sum_{j}^{i}\sum_{k}^{n}b_{\mathrm{jk}}(i),j\neq k\neq i\;\text{and}\;j<k$$

(3) $$C_{\mathrm{RBi}}=\frac{2C_{\mathrm{ABi}}}{n^{2}-3n+2},0\leq C_{\mathrm{RBi}}\leq 1$$

## 3 Visual analysis of agricultural veterinary education research based on text mining

### 3.1 Co-occurrence network analysis of keywords in agricultural veterinary education research

**Figure 3:**
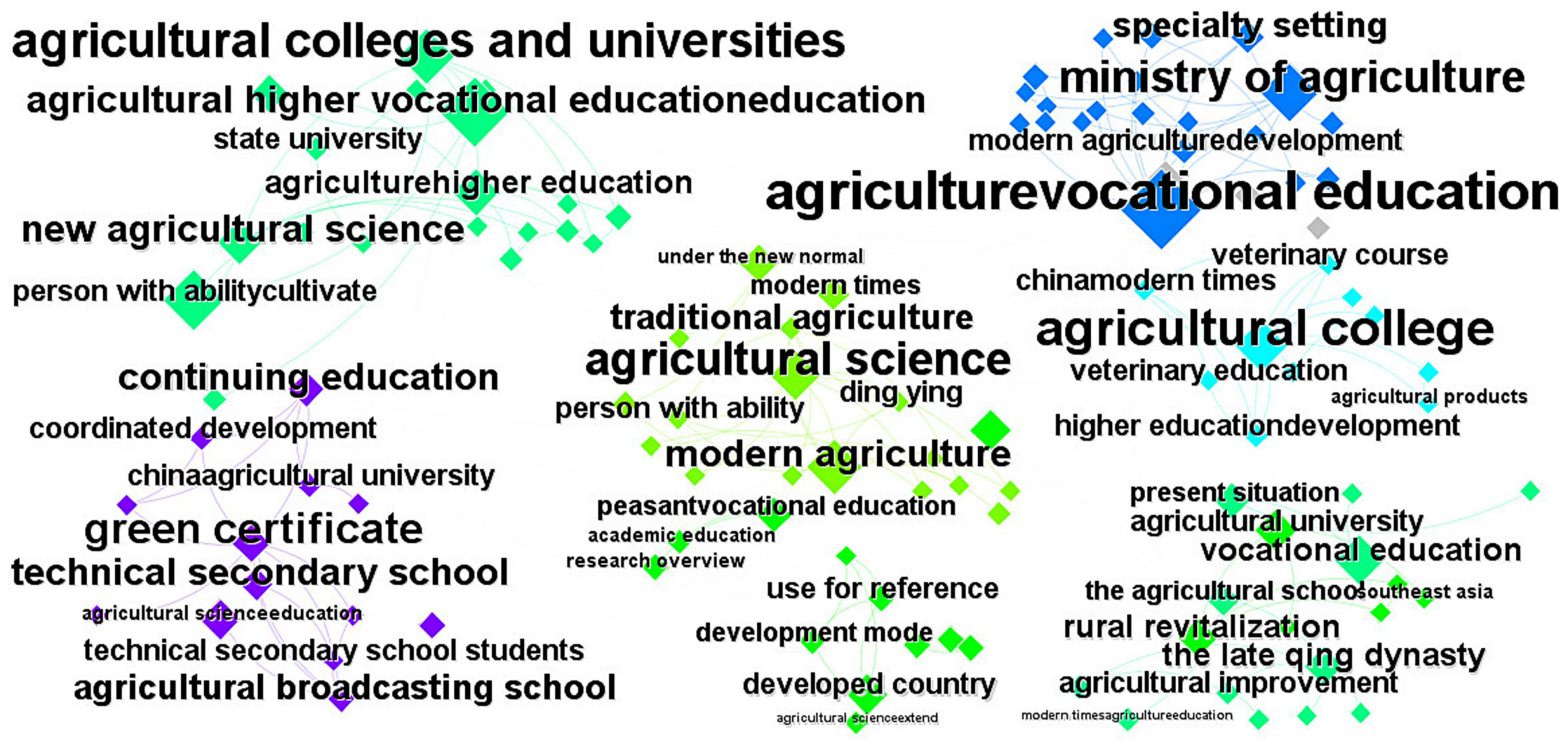
Co-occurrence network of keywords in CNKI.

This paper analyzed and derived a keyword co-occurrence network by conducting text mining on relevant literature. This effectively revealed the main topics and research directions in agricultural veterinary education (Figure 3).

The first cluster (“agricultural colleges and universities,” “agricultural higher vocational education,” and “healthcare personnel cultivation”) highlights the critical role of agricultural veterinary colleges in cultivating professional talent. The second cluster (“continuing education” and “technical secondary school”) indicates that agricultural veterinary education not only cultivates high-level talent but also enhances farmers’ medical knowledge through continuing education. For example, through “green certificate” certification, farmers can gain higher agricultural veterinary service capabilities and professional qualifications. “Agriculture vocational education” is the core keyword in cluster three, demonstrating how agricultural veterinary education meets the needs of modern agriculture and veterinary services. Furthermore, the keyword “specialty setting” indicates the scientific and rational nature of agricultural veterinary specialty settings. This emphasizes that agricultural veterinary education must be integrated with agricultural development trends to ensure the cultivation of professional talents adapted to the needs of modern agriculture. Cluster four (“under the new normal”) reveals the new socioeconomic context facing agricultural veterinary education. In this new era, agricultural veterinary education must focus on cultivating agricultural veterinary professionals with interdisciplinary skills to promote the integrated development of agriculture and healthcare. Cluster five (“development mode,” “agricultural science extend”) indicates that agricultural veterinary education is moving toward a more comprehensive and interdisciplinary direction. Agricultural veterinary education must continually expand its disciplinary scope and training pathways to enhance its influence in livestock production security, disease prevention, and control. The keywords “Veterinary course” and “veterinary education” are part of cluster 6, highlighting the significance of veterinary medicine in agricultural veterinary education. Veterinary medicine plays a crucial role not only in the sustainable development of agriculture but also in safeguarding public health, primarily through the prevention of agricultural pests and the control of animal diseases. Cluster seven (“rural revitalization”) indicates that agricultural veterinary education is closely linked to the national rural revitalization strategy. Rural revitalization requires improvements in agricultural production and the enhancement of rural veterinary care. Strengthening agricultural veterinary education can effectively support the implementation of the rural revitalization strategy and promote the joint development of modern agriculture and agricultural veterinary medicine.

**Figure 4:**
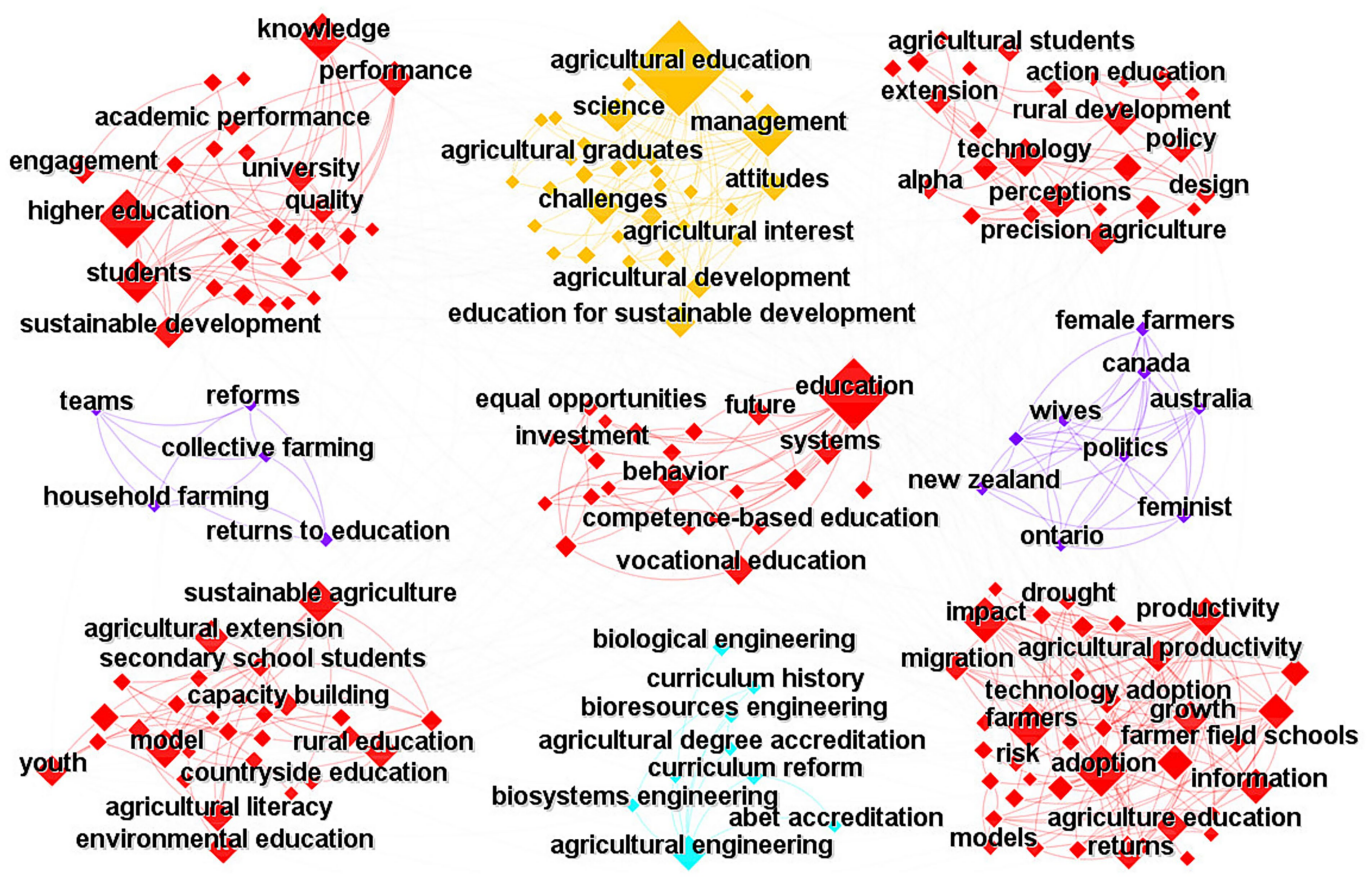
Co-occurrence network of keywords in WoS.

Using the same method, a co-occurrence network analysis of keywords in the WoS agricultural veterinary education research (Figure 4) revealed that the keywords can be roughly divided into nine clusters. The first cluster (“higher education,” “students,” “academic performance,” and “quality”) highlights the quality of students in higher agricultural veterinary education, including aspects such as academic performance. The second cluster (“science,” “management,” and “agricultural interest”) highlights the application of scientific management in agricultural veterinary education, emphasizing the cultivation of students’ interest in agricultural veterinary medicine. The third cluster (“agricultural students,” “action education,” and “policy”) reflects the critical role of agricultural veterinary education policy in promoting agricultural veterinary education. It emphasizes the importance of practical learning for veterinary students in the field of agriculture. The fourth cluster (“reforms”) highlights the need for reform in agricultural veterinary education. The fifth cluster (“equal opportunities”) highlights the role of agricultural veterinary education in creating equal educational opportunities for students. The sixth cluster (“female farmers,” “feminist”) indicates that women are a vital force in agricultural production, and agricultural veterinary education should prioritize their participation. The seventh cluster (“rural education,” “agricultural extension”) reflects the critical role played by agricultural veterinary extension in rural agricultural veterinary education. The eighth cluster (“curriculum reform,” “biological engineering,” “agricultural engineering”) emphasizes curriculum reform in agricultural veterinary education, particularly in the areas of agricultural veterinary medicine and bioengineering. The ninth cluster (“farmer field schools,” “technology adoption,” “agricultural productivity”) suggests that agricultural veterinary education should also strengthen farmer training to achieve widespread adoption and greater effectiveness.

From a temporal perspective, the top 15 documents with the highest co-occurrence citation frequency for keywords in WoS-source research literature are primarily concentrated after 1998. Starting in 1998, many studies on agricultural veterinary education emerged. Around 2000, the emergence of keywords such as “Management,” “Farmers,” “Education,” and “Sustainable Agriculture” indicated a growing focus on sustainable agriculture in agricultural veterinary education, with a particular emphasis on the importance of management in this field. Starting in 2011, the issue of higher education in agricultural veterinary education gradually attracted attention. In 2016, the emergence of keywords such as “Productivity,” “Knowledge,” and “Technology” indicated that contemporary agricultural veterinary education places greater emphasis on cultivating scientific and technological agricultural knowledge, aiming to improve agricultural productivity.

### 3.2 Co-citation network analysis of agricultural veterinary education research

Co-citation analysis refers to the situation where authors jointly cite a published article or when a single author is mentioned multiple times. WoS data sources encompass all references of an article, enabling the conduct of co-citation network analysis. The number of articles cited by authors varies across different networks, while the time distribution within the same network tends to be relatively similar. From the perspective of time, the top three scholars are analyzed. The first is scholar Opara LU, whose paper has the most co-citations. This paper primarily focuses on the research prospects of agricultural engineering education in agricultural veterinary education and has been cited and received attention from scholars (23). The paper was published by scholar Ramesh P, who ranked second, and studied the professional capabilities of teachers in higher agricultural veterinary education in India (24). The third author, Jones K, analyzed the relationship between agricultural veterinary education and profession in four countries (25).

## 4 The social network analysis of agricultural veterinary education literature

**Figure 5:**
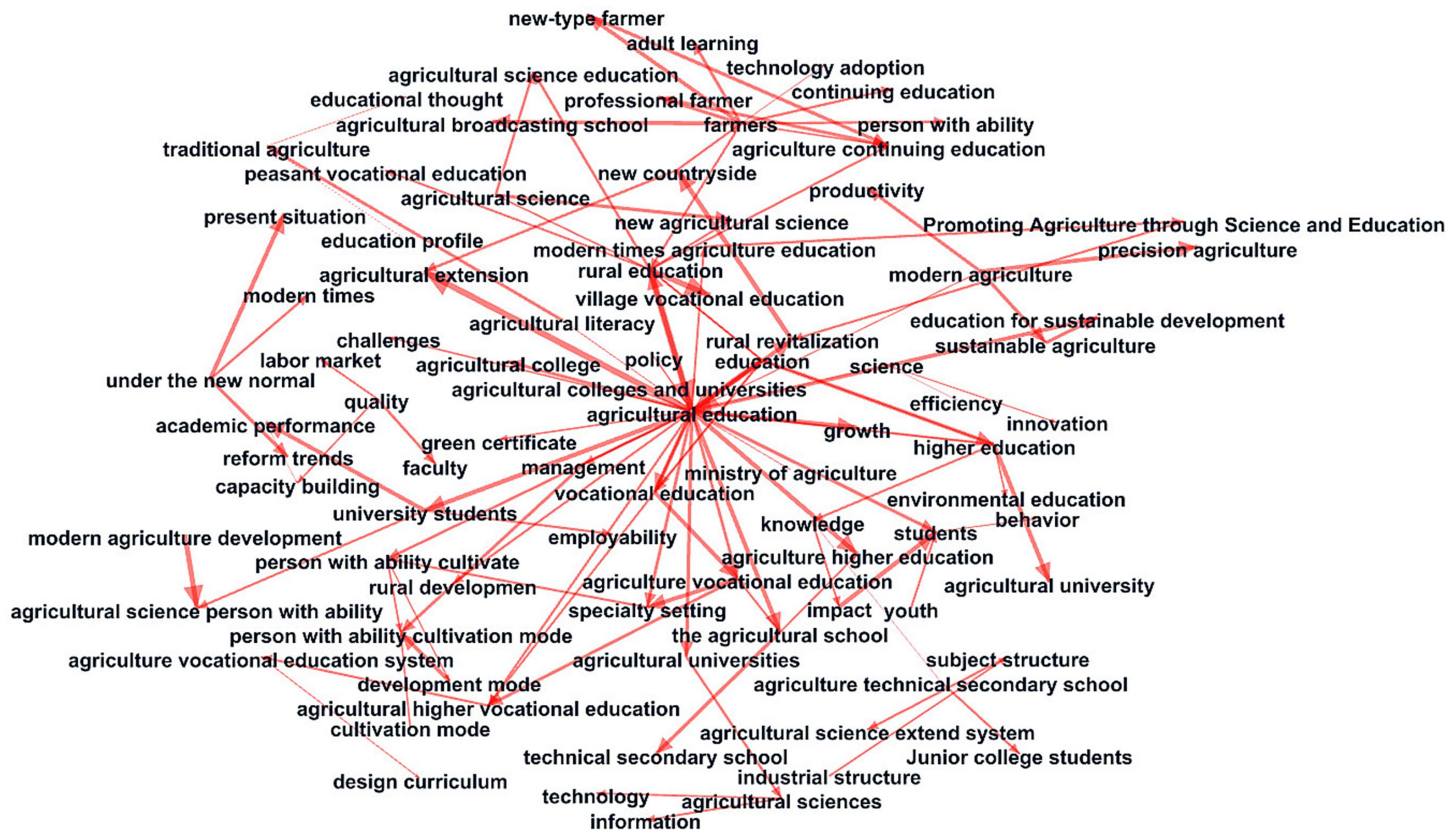
Social network of research literature keywords.

Section 3 of this paper primarily employs text mining and knowledge graph methods to analyze the author-institutional collaboration network, keyword co-occurrence network, and co-citation network in the context of agricultural veterinary education research. The analysis was primarily based on the frequency of literature collaboration, co-occurrence, and co-citation. This article innovatively employs social network analysis, primarily by constructing co-occurrence networks, also known as word-to-word association networks, to calculate the in-degree and out-degree of words. After the calculations are completed, the data is plotted using Gephi software (Figure 5).

From this calculation, it can be observed that “agricultural education” has the highest betweenness centrality, which is highly consistent with the research theme of this project. Next in centrality are “rural health education,” “education,” “farmers’ health,” and “vocational health education.” Looking at the entire social network graph, the closer a node is to the center of the graph, the higher its betweenness centrality. This indicates the high degree of dependence of other nodes on that node and reflects the focus and core of this research. The social network graph also enables the analysis of relationships between different nodes, allowing for a further division of clusters into different emphases based on these relationships. Social network analysis reveals that “agricultural veterinary education” is at the core of the network. This has led to the development of multiple fields, including agricultural vocational veterinary education, agricultural veterinary secondary schools, agricultural veterinary universities, and higher education. This demonstrates that agricultural veterinary education encompasses the primary, intermediate, and advanced levels of the field. Furthermore, the government should provide training to farmers and grassroots veterinarians, imparting knowledge on animal disease prevention and control, as well as breeding techniques. This will help improve their ability to cope with challenges in livestock production and promote the dissemination and application of advanced breeding technologies and management experience. Currently, within the broader context of rural revitalization in China, many research institutions and schools have achieved remarkable results by supporting agricultural production in underdeveloped areas. The development of agricultural veterinary education depends on the continuous promotion by higher education institutions and the training of a large number of practical agricultural professionals by agricultural vocational schools. The core goal of agricultural veterinary education is to cultivate professionals with expertise in agricultural veterinary techniques. Therefore, the competence and performance of agricultural veterinary professionals should be prioritized. The training model of agricultural veterinary schools is relatively rigid, and much room for improvement remains. This is necessary to meet the demands of the agricultural veterinary talent market and is also the only path for agricultural veterinary schools to continue developing and innovating. Future agricultural development will shift toward precision agriculture and transformation, necessitating a growing number of agricultural professionals with expertise in veterinary agricultural techniques.

## 5 Analysis of agricultural veterinary education research domestically and internationally

### 5.1 Analysis of agricultural veterinary education research in different countries

**Figure 6:**
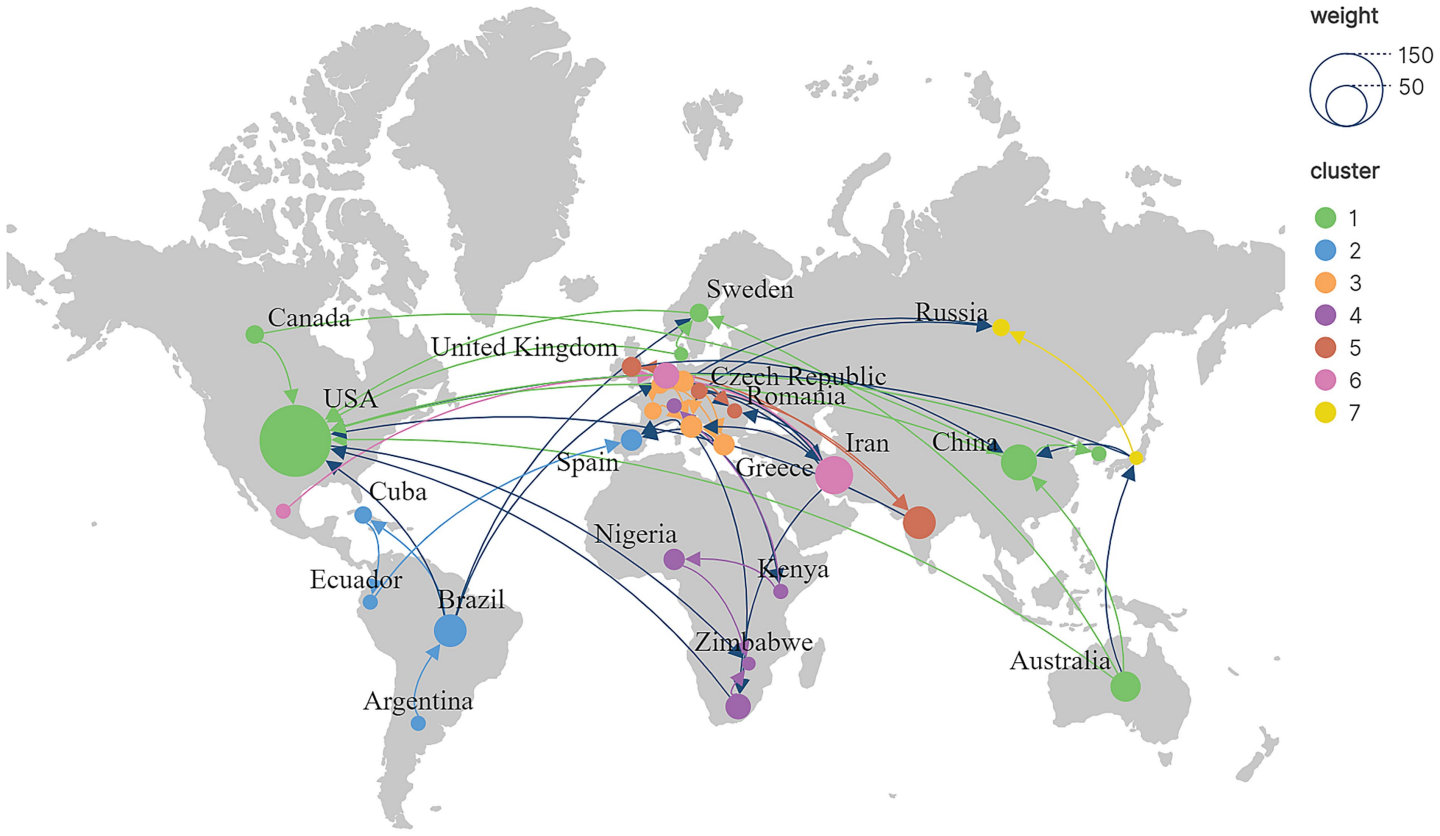
Geographical visualization of agricultural veterinary education research.

From a global perspective (Figure 6), research on agricultural veterinary education is mainly concentrated in North America, Asia, Oceania, and Europe. The United States and Canada represent North America, China and Japan represent Asia, Australia represents Oceania, and Europe is represented by Switzerland and Ukraine. At the same time, countries with more concentrated research groups also tend to have closer cooperation, particularly the United States, China, Australia, and Switzerland. In the United States, the role of agricultural veterinary education in preventing farm work hazards was explored (26). Additionally, the issue of female farmers participating in agricultural veterinary education has also garnered attention (27). The National Association of Landswomen in the United Kingdom supports women’s agricultural and horticultural education (28). In China, to narrow the urban–rural gap, the government has implemented measures to enhance the educational level of farmers, thereby improving agricultural productivity (7). For example, promoting agricultural veterinary technology helps farmers regulate the use of agricultural chemicals (29). As a country with a large population, agriculture is a basic industry in India. However, agricultural veterinary education in India still fails to meet social needs, and there is a need to establish a more refined agricultural veterinary education system (30). At the same time, India’s agricultural higher education has not yet applied marketing concepts, resulting in its lack of competitiveness (31). The Indian government has incorporated environmental sustainability into agricultural, veterinary, and higher education, aiming to provide high-quality talents in the agricultural field (32). In Turkey, agricultural machinery education is primarily conducted through vocational schools to train agricultural machinery technicians (33). However, there is some confusion between agricultural engineering education and agricultural science education in Turkey, and the distinction between the two needs clarification (34). In the context of the Fourth Industrial Revolution, the Mexican government has formulated relevant policies to promote agricultural veterinary education reform; however, it also faces enormous challenges (35).

The most critical factors in the global agricultural veterinary education system include schools, teachers, and students. In Brazil’s agricultural veterinary high schools, the education department integrates neoliberalism and corporate agendas into the curriculum to achieve competitive production (36). Regarding agricultural veterinary teachers, the teaching and research staff of Moldova’s agricultural higher education institutions are facing an aging problem. The reason is that science and education have failed to attract the younger generation. Therefore, improving teachers’ wages and benefits can effectively promote their work enthusiasm (37). The Netherlands has systematically trained teachers of agricultural veterinary education, and schools have also developed active on-the-job training programs to improve the professional level of teachers (38). Regarding agricultural veterinary students, Greece has continuously expanded agricultural veterinary schools and promoted practical education. Agricultural veterinary education has become an essential tool for national and social integration; however, the employment prospects of agricultural veterinary students remain concerning (39). Additionally, farmers are a key target audience for agricultural veterinary education. The Czech Republic offers agricultural veterinary training to farmers through winter schools, which gradually develop into apprenticeship-based vocational education, and subsequently expand into comprehensive education, including agricultural tourism and environmental management (40). Therefore, countries’ agricultural veterinary education systems worldwide are not static, but are adjusted with the changing national development needs.

### 5.2 Research analysis on the significance of promoting agricultural veterinary education

In recent years, the role of agricultural veterinary education has become a topic of considerable interest in the academic community. The vast majority of scholars have affirmed the positive impact of agricultural veterinary education on agricultural development and the development of related rural industries. First, investments in agricultural veterinary technology and education directly promote the growth of the agricultural economy. Based on the panel data of Zhejiang Province, a study using modern econometric methods found that the investment in agricultural veterinary education research funds significantly promoted the development of the agricultural economy (41). Expanding the research scope to 2002 developing countries and emerging economies, the results showed that school education investment had a significant impact on agricultural productivity. In particular, it was crucial for improving animal disease control and ensuring the safety of agricultural production (42). Secondly, investment in agricultural veterinary education also directly increased farmers’ income levels and improved farmers’ labor efficiency and quality of life (43). Taking Guanghan County, Sichuan, China as an example, with the support of a favorable agricultural policy environment, formal agricultural veterinary education not only assisted livestock farmers and grassroots veterinarians in enhancing their skills in animal disease diagnosis and treatment as well as livestock farming management, but also brought direct economic returns to agricultural production (44). Agricultural veterinary education plays a crucial role in driving agricultural modernization and promoting sustainable rural development.

**Figure 7:**
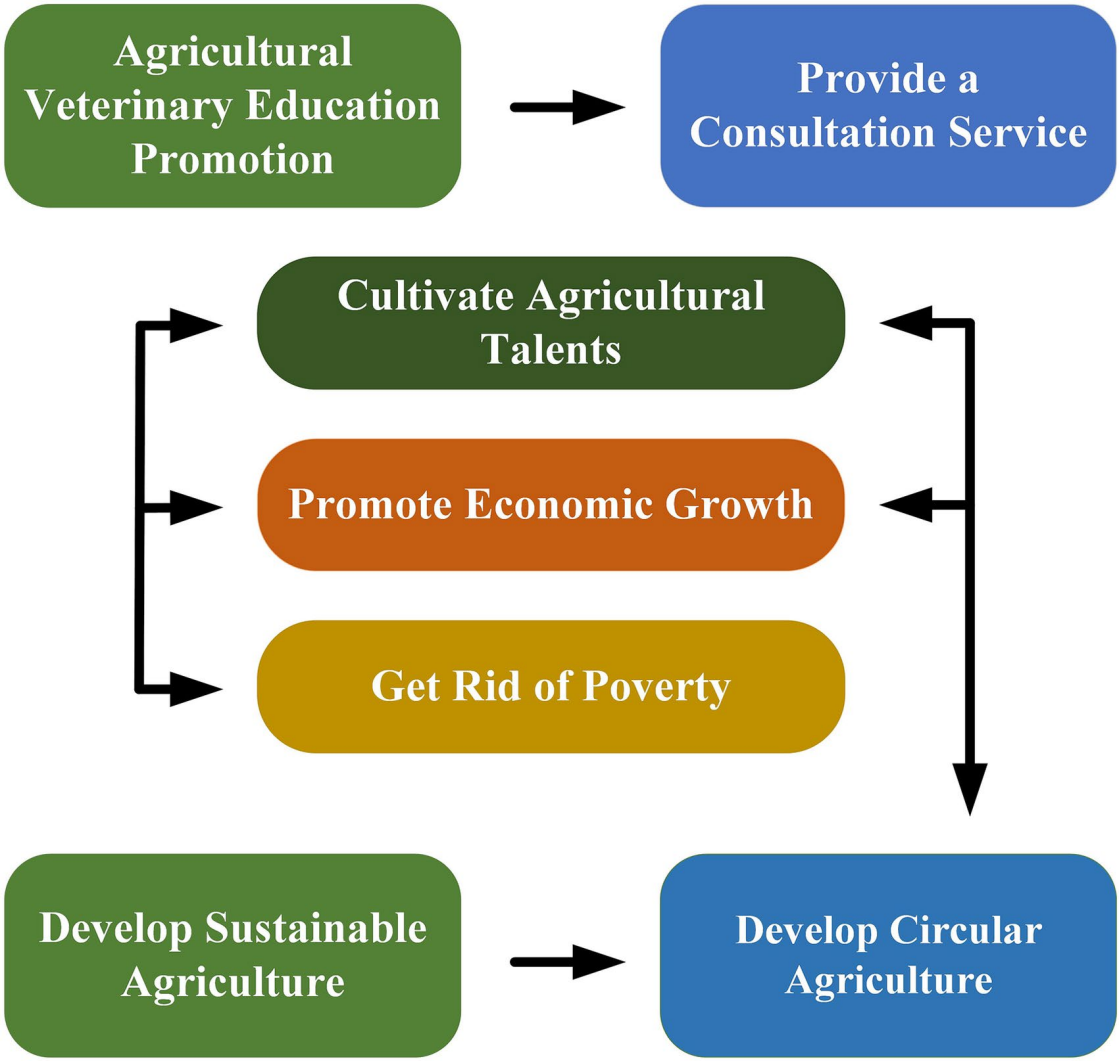
Relationship diagram of the significance of promoting agricultural veterinary education.

Formal agricultural veterinary education mainly includes two contents (Figure 7): one is to provide education, and the other is to provide consulting services. It is the transfer of agricultural veterinary knowledge, which can improve the safety and efficiency of agricultural production (45). Some scholars have noted that when evaluating the impact of agricultural veterinary education on agricultural production performance, other factors, such as producers’ ability to utilize technology, should also be considered (46). Promoting agricultural veterinary education also has a prominent role in reducing farmers’ poverty. In poverty alleviation, China provides agricultural veterinary guidance to poor areas according to local conditions. It promotes the development of related agricultural industry chains. It organizes farmers to learn about kiwifruit, vegetables, and other planting techniques, utilizing advanced veterinary technology in the short term to improve agricultural productivity (47). In addition, in the face of a complex international situation, sustainable agriculture has become a trend in future development, focusing on developing a circular economy rather than agriculture at the expense of the environment. Therefore, agricultural veterinary education has effectively promoted the progress of sustainable agricultural projects and cultivated many agricultural veterinary talents with advanced knowledge and practical ability to maintain sustainable agricultural production (48). However, many young people, especially those in low- and mid-level agricultural veterinary education, have lost interest in the field and are more inclined to choose other industries. This is also the focus of future agricultural veterinary education reform (49).

### 5.3 Agricultural veterinary career and higher education research analysis

Agricultural veterinary education mainly includes vocational education, higher education, and short-term training for farmers. In most countries, agricultural veterinary education is primarily concentrated at the first level. Although vocational and higher veterinary education are aimed at students at different levels, the agricultural veterinary talents they cultivate are indispensable to society. In the study of agricultural vocational veterinary education, Germany employs a dual vocational training system to train farmers on farms, thereby alleviating the increase in unemployment (50). To meet the requirements of sustainable agricultural development, agricultural veterinary technology, vocational education, and training are critical. Iran has systematically implemented education and vocational training standards, capabilities, and educational methods, achieving remarkable results (51). To stimulate young people’s interest in agricultural veterinary careers, it is necessary to incorporate the needs and voices of young people into solutions to improve agricultural veterinary education practices (52).

In the study of agricultural veterinary higher education, the field urgently needs reform to meet the challenges brought about by the global education system’s adjustments (53). Agricultural veterinary education reforms in some countries face challenges in keeping pace with the latest international developments. There is a lack of flexible and innovative teaching methods and curriculum design, particularly in terms of interdisciplinary integration and competency-based teaching systems. In particular, it is essential to recognize the role of agricultural veterinary economics in higher education (54). Some scholars have conducted assessments of teachers and students in agricultural veterinary education to determine whether there is sufficient motivation for thorough reform (55). In the reform of agricultural veterinary education, the first task is to choose a competency-based agricultural veterinary higher education system to ensure the admission of qualified learners and educators (56). Some scholars have specifically studied teachers’ views on higher agricultural veterinary education and proposed practical suggestions for improving teaching quality, including the full utilization of multimedia and smart classrooms for teaching and the regular supervision and provision of feedback on students’ learning (57). In addition to strengthening the theoretical knowledge of agricultural veterinary education, it is also necessary to improve the practical quality of agricultural veterinary education. This provides college students with a wealth of practical agricultural veterinary internship positions, effectively promoting and improving students’ comprehensive quality.

### 5.4 Analysis of agricultural veterinary education methods research

Agricultural veterinary education is often viewed as a model that primarily provides education and training to farmers, which makes agricultural veterinary schools less attractive to students (58). However, as an essential part of rural and agricultural development, agricultural veterinary education must be vigorously developed and provide rural areas with a knowledge-based science and education system (59). On the one hand, various agricultural veterinary education extension methods offer farmers direct training and technical guidance. According to relevant research, agricultural veterinary technology extension has effectively reduced farmers’ use of pesticides in vegetable production (6). On the other hand, in the study of educational methods in agricultural veterinary schools, the mentality of agricultural veterinary students should be given attention, primarily through problem-oriented teaching to stimulate students’ potential (60). Additionally, active learning is also crucial, and off-campus training and cognitive learning can further support this (61). Finally, agricultural veterinary education methods should also focus on students’ needs and be guided by students’ demands for agricultural veterinary education. For example, some majors in agricultural veterinary education focus on agriculture, while a few are combined with engineering. This leads students to prefer engineering-related courses rather than agriculture, which is one of the reasons why this field has difficulty attracting students (62). In a survey of the needs of agricultural veterinary students, the most urgent need among students was for content related to family farming. At the same time, the demand for education on “cash crops” was relatively strong (63). In summary, scientific and reasonable agricultural veterinary education methods should be formulated based on the needs of agricultural veterinary students at different levels and the society’s demand for agricultural veterinary talents, thereby improving the quality of students’ agricultural veterinary education training.

### 5.5 Analysis of strategies for the development of agricultural veterinary education

Agricultural veterinary education has gradually become a necessary stage for farmers. This provides a pathway for young people to become professional farmers and has become a manifestation of agricultural veterinary education that promotes employment (64). As sustainable agriculture becomes a global development trend, agricultural veterinary education plays an increasingly important role in promoting the integration of technology, human resources, and natural resources. In particular, agricultural veterinary education is crucial for alleviating poverty and hunger in some impoverished regions (65). Agricultural veterinary education and training in sub-Saharan Africa face enormous challenges, and increasing attention is being paid to comprehensive systems and sustainable agricultural veterinary development (66). To this end, agricultural veterinary education must be strengthened to improve students’ employability and labor productivity and enhance the benefits of the agricultural veterinary field (67). Globally, Australia has employed a multi-objective approach to agriculture as a guiding framework for establishing universities, focusing on cultivating students who can adapt to market needs and effectively respond to new challenges in the field of agricultural veterinary medicine (68). With the rapid development of science and technology, remote agricultural veterinary education has become widely used, overcoming the limitations of time and space. Students can learn anytime and anywhere, providing more learning opportunities and promoting interaction and communication between teachers and students (69). To improve the quality of agricultural veterinary education, scholars have proposed the scientific evaluation of teacher and student performance, especially in cultivating innovation and technological capabilities. For example, predicting students’ performance in innovation and entrepreneurship education through the neural network (ELM-NN) method can help students improve their self-motivation and learning efficiency (70). In addition, the evaluation of social science teaching effectiveness in agricultural veterinary universities is also a key link, and their comprehensive abilities are evaluated based on their grades and self-assessment scores (71). Finally, in addition to strengthening the internal management of agricultural veterinary education institutions, educational cooperation and exchanges between different countries should be strengthened. In particular, complementarity in educational infrastructure, management experience, and educational content should be strengthened to promote innovation and cross-border development of agricultural veterinary education (72). The Mexican Agricultural Research and Education Center utilizes Twitter to exchange and disseminate information, thereby accelerating the development and popularization of agricultural veterinary education. In the future, agricultural veterinary education and training will become more intelligent and modern, with digital capabilities emerging as a new symbol of excellence in agricultural veterinary education (73).

## 6 Conclusion, limitations, and future perspectives

### 6.1 Research conclusions

This study aimed to identify the progress and future trends in agricultural veterinary medicine education research, providing a reference for government policymakers on agricultural veterinary medicine education. Focusing primarily on agricultural veterinary medicine education research, this study comprehensively analyzed published literature in both Chinese and international publications. Using text mining, knowledge graphs, and social network methods, the study analyzed author and institutional collaboration networks, keyword co-occurrence networks, and co-citation networks. This study compared differences between Chinese and international research on agricultural veterinary medicine education, providing insights into current research progress and potential future issues. This study conducted an in-depth analysis of the literature using various quantitative research methods. This approach provides a new perspective and a more comprehensive analytical framework for literature reviews. Furthermore, the data was sourced from authoritative international databases, and all literature was rigorously screened to ensure quality and relevance, thus ensuring high confidence in the findings. Furthermore, this study creatively employed social network analysis methods to examine the logical relationships between keywords related to agricultural veterinary medicine education in China and abroad. In summary, agricultural veterinary medicine education research primarily focuses on promoting agricultural veterinary medicine education, vocational and higher education in agriculture, practical educational methods, and development strategies for agricultural veterinary medicine education.

The research found that: (1) Research topics in agricultural veterinary education mainly focus on four aspects: promotion of agricultural veterinary education, agricultural vocational and higher education, teaching methods in agricultural veterinary education, and development strategies for agricultural veterinary education. These topics vary significantly in different regions in terms of research level and focus. (2) In terms of research strength, the main body of research in agricultural veterinary education is concentrated in agricultural and veterinary colleges and universities, with less participation from interdisciplinary and non-agricultural research institutions. Research on agricultural veterinary education in China primarily focuses on macro-level policies, institutional development, and connections with national strategies, such as rural revitalization and agricultural security. Other countries focus more on micro-level educational practices, such as improving the professional literacy of farmers and grassroots veterinarians and cultivating practical skills. (3) Although research on agricultural veterinary education has formed a relatively systematic research framework, some issues have not yet been fully explored. For example, there is a lack of research on agricultural veterinary medicine education in junior and senior high schools. The challenges faced by agricultural veterinary medicine education, policies, and the relationship between agricultural veterinary medicine education and other economic and social fields are all worthy of further in-depth discussion.

### 6.2 Limitations

This paper explores the progress and trends in agricultural veterinary education research, filling a gap in the literature review process. However, it still has shortcomings. First, the data in this study mainly focuses on Chinese and English publications, with limited coverage of literature in other languages, which to some extent reduces the generalizability of the conclusions. Second, the paper uses a variety of methods to process the data, but all have certain limitations. Text mining semantic understanding is not accurate enough, knowledge graph entity relationship identification may be incomplete, and social network analysis is insufficient in exploring deep academic influence and knowledge dissemination mechanisms. These will limit the understanding of the current research status and trends. Finally, the internal connections and interaction mechanisms of various research themes are not explored in depth, lacking analysis of their internal relationships, making it difficult to grasp the overall context. At the same time, there is insufficient research on educational differences and adaptability in different regions and socioeconomic backgrounds, and special needs and development paths are not fully considered.

### 6.3 Future perspectives

Regarding data collection, the scope of data sources should be expanded. Relevant research findings in other languages should be actively incorporated, especially literature from regions and countries with unique contributions and rich experience. In terms of research methods, existing methods should be continuously improved and innovated to overcome their limitations. Complex network theory and econometric methods should be combined to deeply analyze issues such as knowledge dissemination paths and the formation mechanisms of academic influence within academic networks. Regarding research content, research should be strengthened on the intrinsic connections and interaction mechanisms among various thematic areas of agricultural veterinary education. Particular attention should be paid to agricultural veterinary medicine education outreach programs for grassroots farmers, as these are effective measures for agricultural development. Specifically, vocational education should keep pace with the times and incorporate modern agricultural concepts, such as eco-friendly, circular, and organic farming, into agricultural veterinary medicine education curricula. In addition to focusing on agricultural, veterinary, vocational, and higher education, emphasis should also be placed on agricultural veterinary medicine education at the junior and senior high school levels. Improve the quality of agricultural veterinary medicine education, promote reform in agricultural veterinary medicine education, and cultivate more professional agricultural veterinary medicine educators. Furthermore, the government should emphasize the importance of agricultural veterinary medicine education and increase the impact of agricultural veterinary medicine schools. This will create more employment opportunities and offer superior remuneration for students pursuing a career in agricultural veterinary medicine.

## Data Availability

Publicly available datasets were analyzed in this study. This data can be found here: https://doi.org/10.6084/m9.figshare.24324034.v1.

## Footnotes

1 Legal text source: https://www.pkulaw.com/law?isFromV5=1
